# Znhit1 and HIF-2α are correlated with cancer stem cell markers in breast cancer patients

**DOI:** 10.1038/s41598-022-18133-8

**Published:** 2022-08-17

**Authors:** Samia A. Ebeid, Nadia A. Abd El Moneim, Sanaa A. El-Benhawy, Rabie Ramadan, Samah E. Ismail

**Affiliations:** 1 Department of Applied Medical Chemistry, Medical Research Institute, Alexandria University, Alexandria, Egypt; 2 Department of Cancer Management and Research, Medical Research Institute, Alexandria University, Alexandria, Egypt; 3 Department of Radiation Sciences, Medical Research Institute, Alexandria University, Alexandria, Egypt; 4 Department of Experimental and Clinical Surgery, Medical Research Institute, Alexandria University, Alexandria, Egypt

**Keywords:** Genetics, Epigenetics, Cancer, Breast cancer, Stem cells, Cancer stem cells, Biomarkers, Diagnostic markers

## Abstract

Epigenetic alterations have emerged as fundamental players in development and progression of breast cancer (BC). A hypoxic tumour microenvironment regulates the stemness phenotype in breast cancer stem cells (BCSCs). The aim of this study was to investigate Znhit1 and HIF-2α gene expression in breast cancer tissues as well as their relation to CSCs markers LGR5, ALDH1A1 and β-catenin in tissue and serum of BC patients. The present study included 160 females divided into two groups, group I: 80 healthy females served as control group and group II: 80 breast cancer patients. Gene expression of tissue Znhit1 and HIF-2α was determined by qRT-PCR. Tissue and serum ALDH1A1, LGR5 and β-catenin levels were determined by ELISA. We found that gene expression of Znhit1 was significantly downregulated in BC tissues. Moreover, it was significantly negatively correlated with clinical stage and β-catenin levels in BC patients. Regarding HIF-2α, gene expression of HIF-2α was significantly upregulated in BC tissues. Moreover, it was significantly positively correlated with Her-2/neu expression and β-catenin levels in BC patients. Based upon our results, Znhit1 and HIF-2α may serve as novel therapeutic targets for BC therapy. Additionally, each of serum ALDH1A1, LGR5 and β-catenin may play a crucial role in non-invasive detection of BC with a high specificity and sensitivity.

## Introduction

Breast cancer accounts for 24.5% of all cancers diagnosed in females and it is the first leading cause of cancer-related mortality in females worldwide^1^. Epigenetics is the study of heritable changes in gene expression that do not affect primary DNA sequence^2^. Failure of the proper maintenance of heritable epigenetic marks can lead to abnormal induction or suppression of several signaling pathways and results in many diseases including cancer^3^. Zinc finger HIT-type containing 1 (Znhit1), is a subunit of sucrose non-fermenting 2 (Snf2)-related cyclic adenosine monophosphate response element binding protein-binding protein (CBP) activator protein (SRCAP) complex, which specifically can alter chromatin structure by promoting the inclusion of H2AZ-H2B dimers into nucleosomes^4^. Znhit1 is overexpressed in response to genotoxic stresses and activating transcription of numerous p53 target genes^5^. However, the function of Znhit1 in cancer remains largely unknown.

A hypoxic tumour microenvironment regulates the stemness phenotype in breast cancer stem cells (BCSCs). The activation of Hypoxia-inducible factors (HIFs) is one of the fundamental mechanisms of stemness maintenance triggered by hypoxia^6^. HIFs are made up of a heterodimer of an O2-regulated α subunit (HIF-1α, HIF-2α and HIF-3α) and a constitutively expressed β subunit^7^. Despite their great homology, HIF-1α and HIF-2α regulate diverse target genes and have different roles in invasion, proliferation and chemotherapy resistance^8^.

Accumulating evidence suggests that the presence of a small number of immature cells, known as BCSCs appears to be the most likely cause of tumour progression, metastasis and drug resistance^9,10^. Aldehyde dehydrogenase 1^+^ (ALDH1^+^) and leucine rich repeat containing G protein coupled receptor 5^+^ (LGR5^+^) are the most commonly used biomarkers to identify the BCSC phenotype^11–13^.

Aldehyde dehydrogenase 1 is an intracellular enzyme catalyzing the oxidation of intracellular aldehydes. It contributes to differentiation of normal and tumour stem cell^14^. ALDH1 proteins consist of 3 main isozymes namely ALDH1A1, ALDH1A2 and ALDH1A3 with ALDH1A1 being the most specific which is largely reported to be related to poor prognosis^15^.

Leucine-rich repeat-containing G protein-coupled receptor 5 is a well-recognized marker for adult SCs as well as CSCs. LGR5 promotes BC progression and CSCs maintenance partially via stimulation of Wnt/β-catenin signaling pathway^16,17^. BCSCs with activated Wnt/β-catenin signaling pathway are substantially more tumorigenic than those without and blocking Wnt/β-catenin pathway inhibits BC metastasis by suppressing BCSCs phenotype^18^. β-catenin is the major mediator of Wnt/β-catenin signaling pathway. It is a multifunctional protein that either associates with cadherins at the cell membrane to regulate cellular adhesion or translocates to the nucleus, where it activates transcription of Wnt target genes^19^.

The aim of this study was to investigate Znhit1 and HIF-2α gene expression in breast cancer tissues as well as their relation to CSCs markers LGR5, ALDH1A1 and β-catenin in tissue and serum of BC patients. Therefore, this study can help to find new target against BC progression and reveal a possible molecular mechanism for the Znhit1and HIF-2α associated regulation in BC, providing innovate therapeutic strategies for the treatment of BC.

## Results

### Znhit1 gene expression in BC tissues

**Figure 1:**
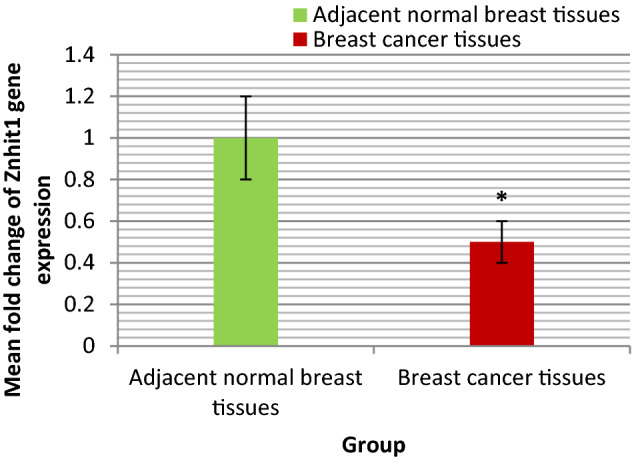
Bar chart representing the mean fold change of Znhit1 gene expression in BC tissues and adjacent normal breast tissues.

The range and mean ± SE fold change of Znhit1 gene expression in BC tissues and adjacent normal breast tissues were shown in Table 1 and illustrated in Fig. 1. The statistical analyses of these results were also shown in Table 1. As presented in Table 1 the mean fold change of Znhit1 gene expression in BC tissues was statistically significantly lower than that in adjacent normal breast tissues (P = 0.009).

**Table 1:** Statistical analyses of Znhit1 and HIF-2α gene expression in BC tissues and adjacent normal breast tissues.

		Adjacent normal breast tissues	Breast cancer tissues
Znhit1	Range	0.02–6.8	0.0004–2.1
	Mean ± SE	1 ± 0.2	0.5 ± 0.1
	P		0.009*
HIF-2α	Range	0.01–4.2	0.001–10.7
	Mean ± SE	1.0 ± 0.2	2.3 ± 0.6
	P		0.02*

*P* P value for comparing between BC tissues and adjacent normal breast tissues.

*Statistically significant difference at P ≤ 0.05.

### HIF-2α gene expression in BC tissues

**Figure 2:**
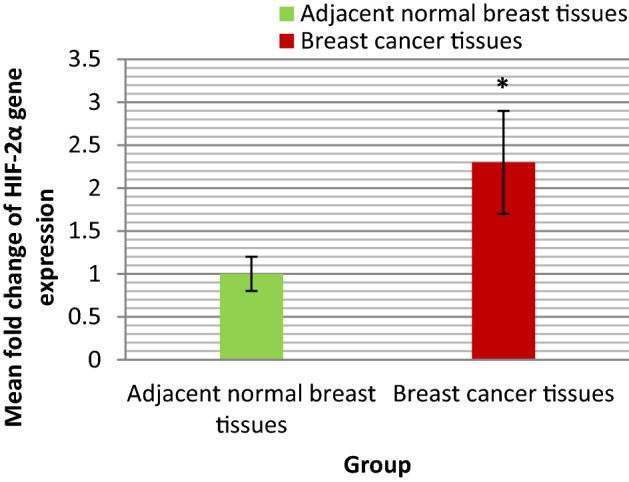
Bar chart representing the mean fold change of HIF-2α gene expression in BC tissues and adjacent normal breast tissues.

The range and mean ± SE fold change of HIF-2α gene expression in BC tissues and adjacent normal breast tissues were shown in Table 1 and illustrated in Fig. 2. The statistical analyses of these results were also shown in Table 1. As presented in Table 1 the mean fold change of HIF-2α gene expression in BC tissues was statistically significantly higher than that in adjacent normal breast tissue (P = 0.02).

### ALDH1A1 levels (pg/ml) in BC tissues

**Figure 3:**
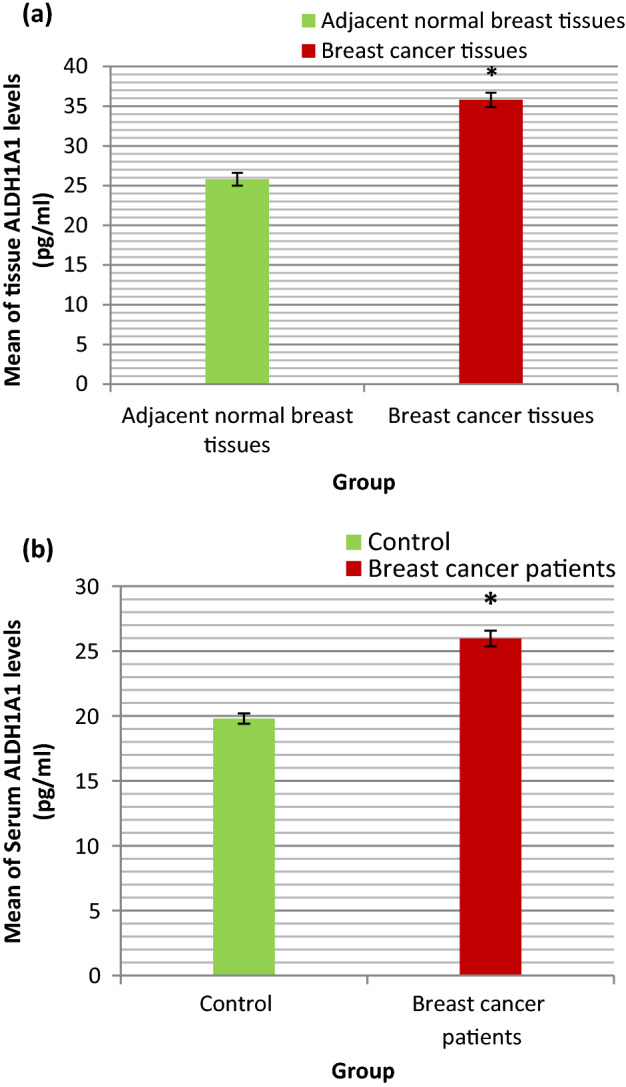
(**a**) Bar chart representing the mean value of ALDH1A1 (pg/ml) in BC tissues and adjacent normal breast tissues. (**b**) Bar chart representing the mean value of serum ALDH1A1 (pg/ml) in control group and BC patients group.

The range and mean ± SE of ALDH1A1 (pg/ml) in BC tissues and adjacent normal breast tissues were shown in Table 2 and illustrated in Fig. 3a. The statistical analyses of these results were also shown in Table 2. As presented in Table 2 the mean value of ALDH1A1 in BC tissues was statistically significantly higher than that in adjacent normal breast tissues (P < 0.001).

**Table 2:** Statistical analyses of ALDHIAI, LGR5 and β-catenin levels (pg/ml) in BC tissues and adjacent normal breast tissues.

		Adjacent normal breast tissues	Breast cancer tissues
Tissue ALDHIAI	Range	21.5–36	25–47
	Mean ± SE	25.8 ± 0.8	35.8 ± 0.9
	P		< 0.001*
Tissue LGR5	Range	32.5–49.5	39–65
	Mean ± SE	38.8 ± 0.8	48 ± 0.9
	P		< 0.001*
Tissue β-catenin	Range	172.5–194.5	178–297
	Mean ± SE	180.7 ± 1.5	228.5 ± 4.9
	P		< 0.001*

*P* P value for comparing between BC tissues and adjacent normal breast tissues.

*Statistically significant difference at P ≤ 0.05.

### ALDH1A1 levels (pg/ml) in serum of BC patients

The range and mean ± SE of serum ALDH1A1 (pg/ml) in control group and BC patients group were shown in Table 3 and illustrated in Fig. 3b. The statistical analyses of these results were also shown in Table 3. As presented in Table 3 the mean value of serum ALDH1A1 in BC patients group was statistically significantly higher than that in control group (P < 0.001).

**Table 3:** Statistical analyses of serum ALDH1A1, LGR5 and β-catenin levels (pg/ml) in control group and BC patients group.

		Control group	Breast cancer patients group
Serum ALDH1A1	Range	17–21	20.5–35.5
	Mean ± SE	19.8 ± 0.4	25.97 ± 0.6
	P		< 0.001*
Serum LGR5	Range	22–30.5	28–46
	Mean ± SE	26.4 ± 0.8	36.2 ± 0.7
	P		< 0.001*
Serum β-catenin	Range	119.5–170	163.5–232.5
	Mean ± SE	137.7 ± 4.7	192.9 ± 3.1
	P		< 0.001*

*P* P value for comparing between control group and BC patients group.

*Statistically significant difference at P ≤ 0.05.

### LGR5 levels (pg/ml) in BC tissues

**Figure 4:**
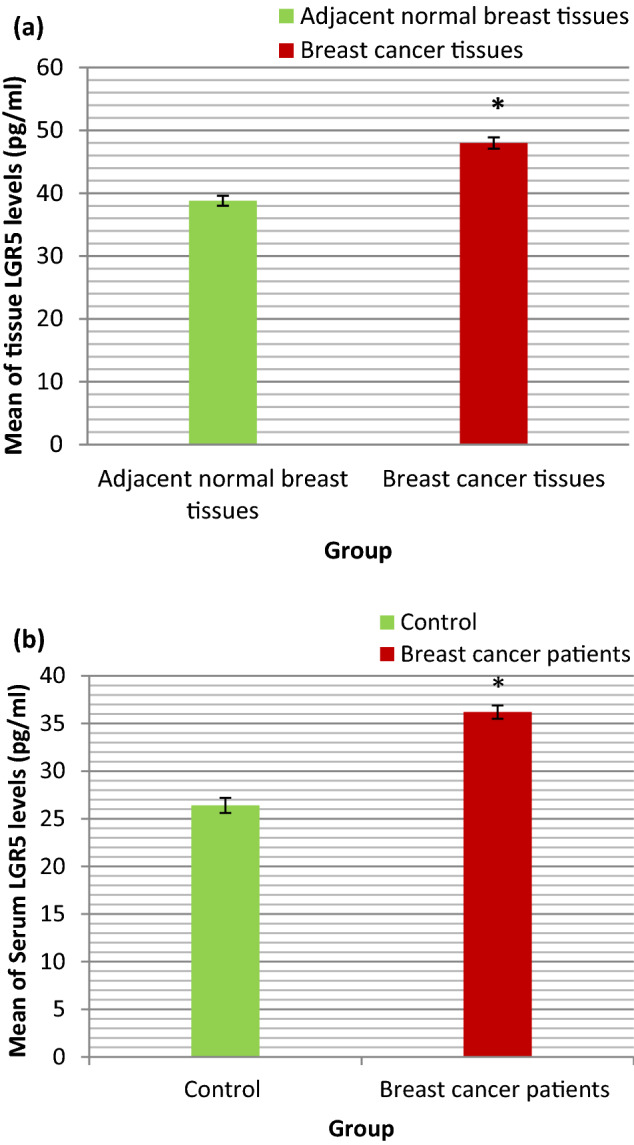
(**a**) Bar chart representing the mean value of LGR5 (pg/ml) in BC tissues and adjacent normal breast tissues. (**b**) Bar chart representing the mean value of serum LGR5 (pg/ml) in control group and BC patients group.

The range and mean ± SE of LGR5 (pg/ml) in BC tissues and adjacent normal breast tissues were shown in Table 2 and illustrated in Fig. 4a. The statistical analyses of these results were also shown in Table 2. As presented in Table 2 the mean value of LGR5 in BC tissues was statistically significantly higher than that in adjacent normal breast tissues (P < 0.001).

### LGR5 levels (pg/ml) in serum of BC patients

The range and mean ± SE of serum LGR5 (pg/ml) in control group and BC patients group were shown in Table 3 and illustrated in Fig. 4b. The statistical analyses of these results were also shown in Table 3. As presented in Table 3 the mean value of serum LGR5 in BC patients group was statistically significantly higher than that in control group (P < 0.001).

### β-catenin levels (pg/ml) in BC tissues

**Figure 5:**
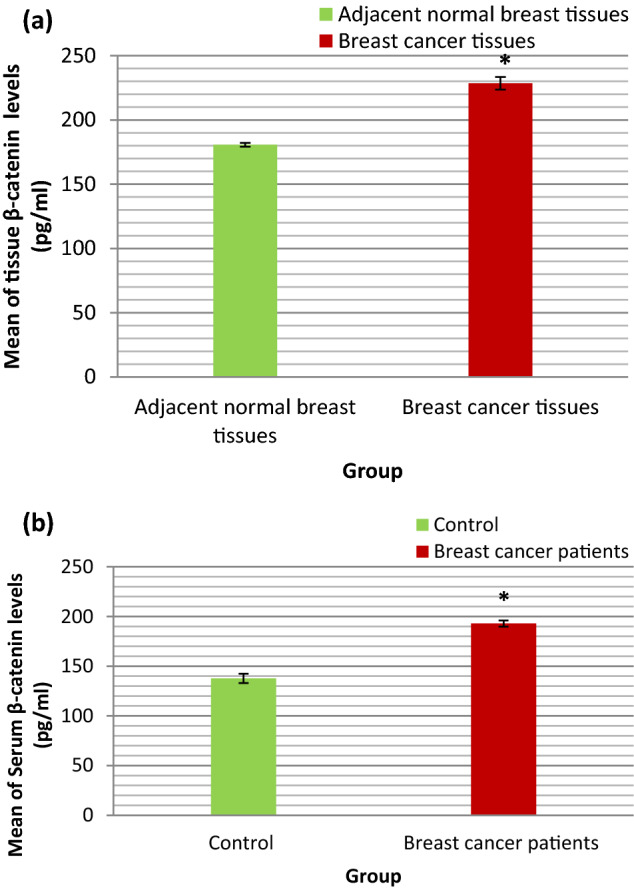
(**a**) Bar chart representing the mean value of β-catenin (pg/ml) in BC tissues and adjacent normal breast tissues. (**b**) Bar chart representing the mean value of serum β-catenin (pg/ml) in control group and BC patients group.

The range and mean ± SE of β-catenin (pg/ml) in BC tissues and adjacent normal breast tissues were shown in Table 2 and illustrated in Fig. 5a. The statistical analyses of these results were also shown in Table 2. As presented in Table 2 the mean value of β-catenin in BC tissues was statistically significantly higher than that in adjacent normal breast tissues (P < 0.001).

### β-catenin levels (pg/ml) in serum of BC patients

The range and mean ± SE of serum β-catenin (pg/ml) in control group and BC patients group were shown in Table 3 and illustrated in Fig. 5b. The statistical analyses of these results were also shown in Table 3. As presented in Table 3 the mean value of serum β-catenin in breast cancer patients group was statistically significantly higher than that in control group (P < 0.001).

### Correlation between different biochemical studied parameters and clinicopathological parameters in BC patients

Znhit1 gene expression was statistically significantly negatively correlated with clinical stage (r =  − 0.336, P = 0.037), HIF-2α gene expression was statistically significantly positively correlated with Her-2/neu expression (r = 0.601, P < 0.001) and LGR5 either in tissue or serum was statistically significantly negatively correlated with PR status (r =  − 0.300, P = 0.044 and r =  − 0.344, p = 0.043; respectively), while there was insignificant correlation between these parameters and the other clinicopathological characters.

### Correlation between different biochemical studied parameters in BC patients

**Figure 6:**
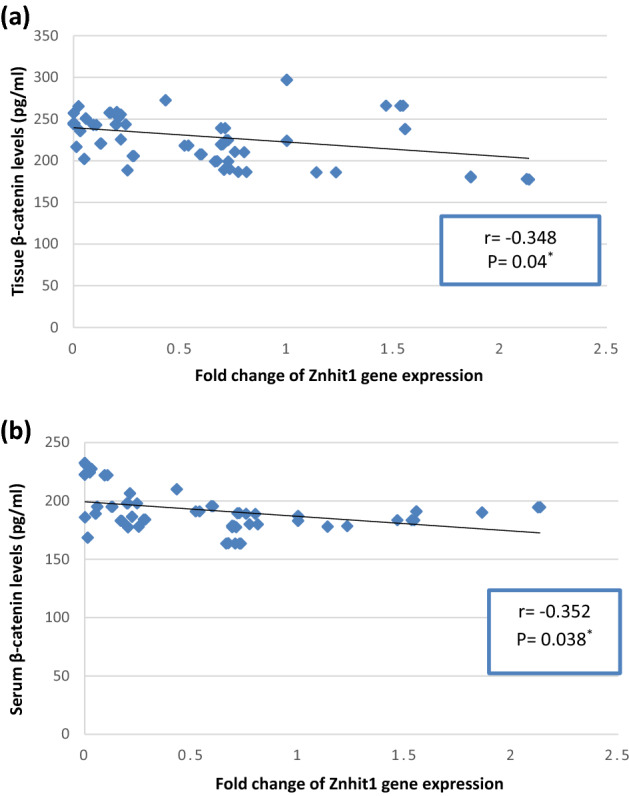
(**a**) Correlation between fold change of Znhit1 gene expression and β-catenin levels (pg/ml) in breast cancer tissues. (**b**) Correlation between fold change of Znhit1 gene expression and serum β-catenin levels (pg/ml) in breast cancer patients.

**Figure 7:**
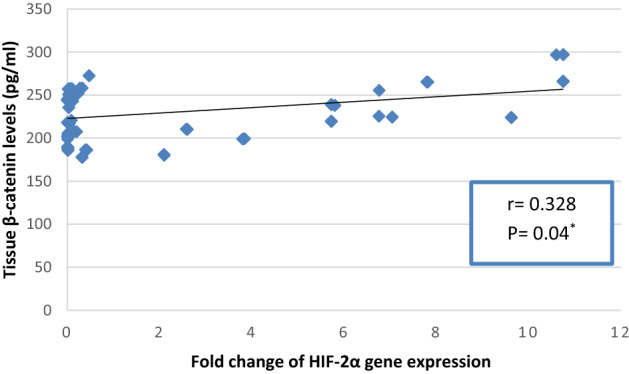
Correlation between fold change of HIF-2α gene expression and β-catenin levels (pg/ml) in breast cancer tissues.

As presented in Fig. 6a,b, Znhit1 gene expression was statistically significantly negatively correlated with tissue and serum β-catenin (r =  − 0.348, P = 0.04 and r =  − 0.352, P = 0.038; respectively), while it was insignificantly negatively correlated with ALDH1A1 and LGR5 levels in tissue and serum of BC patients. As presented in Fig. 7, HIF-2α gene expression was statistically significantly positively correlated with tissue β-catenin (r = 0.328, P = 0.04), while it was insignificantly correlated with serum β-catenin, tissue and serum ALDH1A1 and LGR5. Tissue ALDH1A1 was statistically significantly positively correlated with serum ALDH1A1, tissue LGR5 and tissue β-catenin (r = 0.489, P = 0.003, r = 0.338, P = 0.04 and r = 0.391, P = 0.02; respectively). Serum ALDH1A1 was statistically significantly positively correlated with serum LGR5 and serum β-catenin (r = 0.628, P < 0.001 and r = 0.550, P = 0.001; respectively). Tissue LGR5 was statistically significantly positively correlated with serum LGR5 and tissue β-catenin (r = 0.466, P = 0.005 and r = 0.419, P = 0.012; respectively). Serum LGR5 was statistically significantly positively correlated with serum β-catenin (r = 0.606, P < 0.001). Tissue β-catenin was statistically significantly positively correlated with serum β-catenin (r = 0.397, P = 0.018).

### Diagnostic performance of ALDH1A1, LGR5 and β-catenin in serum of breast cancer patients

**Figure 8:**
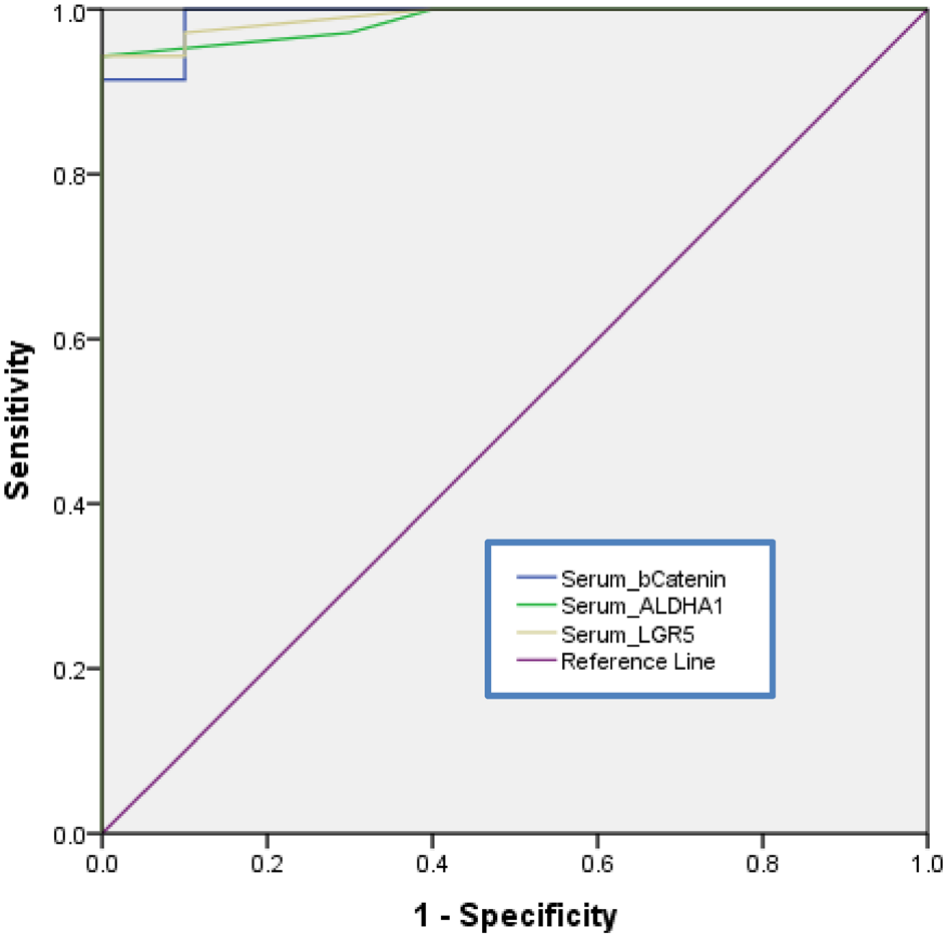
Graphical presentation of the ROC curve for serum ALDH1A1, serum LGR5 and serum β-catenin in breast cancer patients.

The ROC curve analysis was used to compare the diagnostic values of the studied parameters. The higher area under the ROC curve (AUC) corresponds to a better diagnostic test. As presented in Fig. 8, serum ALDH1A1 had significant AUC (0.986, P < 0.001) with 100% specificity and 94% sensitivity at cut off value ≥ 21.5 pg/ml, serum LGR5 had significant AUC (0.99, P < 0.001) with 90% specificity and 97% sensitivity at cut off value ≥ 28.75 pg/ml and serum β-catenin had significant AUC (0.991, P < 0.001) with 90% specificity and 94% sensitivity at cut off value ≥ 166 pg/ml.

## Discussion

The results of our study indicated that the gene expression of Znhit1 in BC tissues was statistically significantly lower than that in adjacent normal breast tissues. Moreover, our results revealed statistically significant negative correlation between gene expression of Znhit1 in BC tissues and clinical stage of patients, suggesting the role of Znhit1 repression in tumor progression. Similarly, Sun et al. demonstrated that expression of Znhit1 gene was significantly decreased in acute myeloid leukemia (AML) patients compared with healthy blood cell samples^20^. Znhit1 has been shown to trigger cell cycle arrest as well as play an important role in controlling cell proliferation^21^. According to Cuadrado et al. study which demonstrated that under normal growing conditions, cyclin G1 stimulates degradation of Znhit1 and can possibly regulate its expression levels^5^, we suggest that cyclin G1 is overexpressed in cancer cells leading to downregulaion of Znhit1 expression.

Moreover, the current study revealed that Znhit1 gene expression in BC tissues was statistically significantly negatively correlated with β-catenin levels in tissue and serum of BC patients. This finding suggests that downregulation of Znhit1 may contribute to induction and maintenance of stemness in BC. To the best of our knowledge, the present study is the first study revealed this correlation. Our explanation of this negative correlation that the downregulation of Znhit1 expression in BC tissues may downregulate Phosphatase and tensin homolog (PTEN) expression along with activation of Phosphoinositide 3-kinase (PI3K)/A serine threonine protein kinase (Akt) signaling pathway^20^, PI3K/Akt activation can stimulate the Wnt/β-catenin signaling pathway via the Glycogen synthase kinase 3 beta phosphorylation by the phosphorylated Akt1/2, preventing β-catenin destruction complex formation^22^.

Regarding HIF-2α, the results of our study indicated that gene expression of HIF-2α in BC tissues was statistically significantly higher than that in adjacent normal breast tissues. Moreover, gene expression of HIF-2α was statistically significantly positively correlated with Her-2/neu expression in human BC tissues. These results were in accordance with in vitro study conducted by Jarman et al. who revealed that Her-2 overexpression in MCF-7 cells leads to upregulation of HIF-2α in normoxia and in response to hypoxia. In addition, they showed that upregulation of Her-2 in this model promotes the expression of many hypoxic response genes both in normoxia and in response to acute (24 h) and chronic (10 weeks) hypoxia, indicating that growth factor signaling may increase the cellular response to hypoxia through HIF-2α^23^. Additionally, our results revealed statistically significant positive correlation between HIF-2α gene expression and β-catenin levels in human BC tissues, suggesting the role of HIF-2α in driving stemness in BC. This finding is consistent with the study of Yan et al. who demonstrated that upregulation of HIF-2α in MCF-7 and MDA-MB-231 BC cells induced stemness and promoted paclitaxel resistance by stimulating the Notch and Wnt signaling pathways. Wnt pathway activation was related to overexpression of β-catenin^24^. To the best of our knowledge, this is the first study showed that HIF-2α expression is related to Her-2/neu expression and the expression of β-catenin in BC patients.

To elucidate the biological significance of ALDH1A1 and its regulation in BC, we examined the ALDH1A1 levels in tissue and serum of BC patients. The results of this study revealed that the ALDH1A1 levels in tissue and serum of BC patients were statistically significantly higher than that in adjacent normal breast tissue and serum of control subjects respectively. Furthermore, our results revealed statistically significant positive correlation between tissue and serum ALDH1A1 levels in BC patients. These findings suggest that serum ALDH1A1 can be used as biomarker for non-invasive detection of cancer stem cells in BC patients. Our results support study conducted by Gyan et al. who examined the ALDH1A1 protein expression in African BC patients by Immunohistochemistry assay. The results of their study demonstrated that protein expression of ALDH1A1 was high in African BC patients^25^. Consistent with our results, Rossi et al. showed that serum ALDH1A1 protein levels are statistically higher in patients with non-small cell lung cancer (NSCLC) compared to control group^26^.

Moreover, our results showed that ALDH1A1 levels were statistically significantly positively correlated with LGR5 and β-catenin levels in tissue and serum of BC patients. These findings suggest that ALDH1A1 expression may be regulated by LGR5 and β-catenin in BC. In line with our findings, Gao et al. demonstrated that the protein expression of ALDH1A1 and LGR5 were significantly related in NSCLC and were correlated with the NSCLC progression and indicated poor prognosis^27^. Cui et al. indicated that Notch and Wnt/β-catenin signaling pathways that are important in drug resistance were aberrantly induced in stem-like ALDH^high^/CD44^+^ cells compared with non-stem-like ALDH^low^/CD44^+^ cells in BC^28^. Furthermore, Sun et al. suggested that ALDH1A1 expression may be regulated by β-catenin in BC^29^. Collectively, these findings suggest that ALDH1A1 levels may help to identify high-risk BC patients and thus can be considered as potential therapeutic and/or diagnostic target.

The results of our study revealed that LGR5 levels in tissue and serum of BC patients were statistically significantly higher than that in adjacent normal breast tissue and serum of control subjects respectively. Moreover, there was statistically significant positive correlation between tissue and serum LGR5 levels in BC patients. These findings suggest that serum LGR5 will be one of the potential candidate biomarkers for non-invasive detection of cancer stem cells in BC patients. Our results were consistent with previous studies evaluated LGR5 protein expression in BC tissues by Immunohistochemistry assay and observed that LGR5 protein expression was lower in normal breast tissue compared to BC tissue^17,30^.

Nevertheless, results of our study showed that LGR5 levels in tissue and serum of BC patients were statistically significantly positively correlated with β-catenin levels. This finding supports the notion that LGR5 activates Wnt/β-catenin pathway in BC patients^17^. This correlation may be explained by recent in vitro study conducted by Chen et al. who demonstrated that a protein kinase A (PKA) activity was increased by LGR5 overexpression and was reduced by suppression of LGR5 in BC cells. Also, expression level of LGR5 and activity of PKA were positively related to activation of β-catenin while negatively related to activation of Glycogen synthase kinase 3 beta in BC cells. These mean that LGR5 stimulates the Wnt/β-catenin pathway in BC cells by activation of PKA^31^. These findings suggest that LGR5 is more of a molecular marker for CSCs but may also promote cancer cell growth and tumor development in BC. Thus, targeting of LGR5 could thus be a possible therapeutic strategy for BC treatment.

The specific role of β-catenin in breast carcinoma remains unclear, results of our study demonstrated that β-catenin levels in tissue and serum of BC patients were statistically significantly higher than that in adjacent normal breast tissue and serum of control subjects respectively. Moreover, there was statistically significant positive correlation between tissue and serum β-catenin levels in BC patients. These suggest that serum β-catenin levels may be used as non-invasive marker to distinguish BC patients from normal controls. These results were consistent with Xu et al. study which demonstrated that the aberrant membranous expression of β-catenin and the positive nuclear/cytoplasmic expression of β-catenin were detected in 43.5% and 78.1% of the BC tissues, respectively^32^. Recent study on BC proposed that deregulation of Wnt/β-catenin pathway may be due to downregulation of its negative regulators. This was supported by the finding that the extracellular inhibitor of Wnt/β-catenin pathway expression named secreted frizzled-related protein 1 (SFRP1) is significantly downregulated in many breast tumors and this is related to poor prognosis as well as therapy resistance^33^.

Our results showed that each of serum β-catenin, LGR5 and ALDH1A1 has high diagnostic efficacy in BC. Similarly, Li et al. study demonstrated that β-catenin levels in serum had high effectiveness in colorectal carcinoma diagnosis and showed that serum β-catenin levels play a role in early detection of colorectal carcinoma^34^. To the best of our knowledge, the diagnostic efficacy of serum β-catenin, LGR5 and ALDH1A1 in BC not yet reported in any literature.

## Conclusion

From our study we can conclude that Znhit1 repression may play a role in tumour progression as well as induction and maintenance of stemness in BC. HIF-2α may play a role in aggressiveness of Her-2-positive BC and driving stemness in BC patients. The elevated levels of β-catenin in BC patients may be due to downregulation of Znhit1 and upregulation of HIF-2α and LGR5 which activate Wnt/β-catenin signaling pathway. Moreover, the elevated levels of β-catenin may lead to upregulation of Wnt target gene ALDH1A1. β-catenin may be a marker of advanced BC and thus may represent a new diagnostic and therapeutic target. Each of serum ALDH1A1, LGR5 and β-catenin may play a crucial role in non-invasive detection of BC with a high specificity and sensitivity.

## Recommendations

Further studies are required to explain the mechanism of Znhit1 downregulation in BC to explore new therapeutic agents that upregulate Znhit1 expression in BC. Targeting of HIF-2α in Her-2-positive BC may be an effective therapeutic intervention. Follow up of BC patients involved in our study is required to investigate the role of studied parameters in BC prognosis.

## Subjects and methods

This study was approved by ethical committee of Medical Research Institute (MRI)-Alexandria University and was conducted on one hundred sixty females. They were divided into two groups, Group I which included 80 apparently normal healthy female volunteers served as control group and group II which included 80 females of matched age and menstrual state as the previous group having breast carcinoma of clinical stage II–III.

All Patients were chosen from those admitted to Experimental and Clinical Surgery Department and Department of Cancer Management and Research, MRI, Alexandria University. A written informed consents were taken from all contributors in this study, in accordance with the Declaration of Helsinki. Patients who were metastatic at the time of diagnosis and those who were receiving neoadjuvant therapy were excluded from this study.

Patients were subjected to preoperative evaluation by fine needle aspiration cytology to detect the presence of malignancy and clinical examination to detect the site of the tumour and the presence of axillary lymph node infiltration. Patients were subjected to surgery (mastectomy or conservative) and a written informed consent was taken from all patients before surgery. Pathological grading of the malignant tumour, tumour size, vascular invasion, ER and PR status and Her-2/neu expression were confirmed. The clinico-pathological characteristics of BC patients are represented in Table 4.

**Table 4:** Clinicopathological characteristics of BC patients group (n = 80).

	BC patients group (n = 80)
**Age**	
Mean ± SD	57.6 ± 9.3 years
Range	(44–77) years
**Histological grade**	
II	64 (80%)
III	16 (20%)
**Clinical stage**	
II	48 (60%)
III	32 (40%)
**Tumor size**	
≤ 5	54 (67.5%)
< 5	26 (32.5%)
**Axillary lymph node involvement**	
Positive	50 (62.5%)
Negative	30 (37.5%)
**ER status**	
Positive	76 (95%)
Negative	4 (5%)
**PR status**	
Positive	76 (95%)
Negative	4 (5%)
**Her-2/neu expression**	
Positive	32 (40%)
Negative	48 (60%)
**Vascular invasion**	
Positive	80 (100%)
Negative	0 (0%)

*ER* estrogen receptor status, *PR* progesterone receptor status, *Her-2* human epidermal growth factor receptor 2.

### Tissue sampling

Human BC tissues and adjacent normal breast tissues were taken from patients who underwent surgical resection and were stored at − 80 °C until used for determination of the Znhit1 and HIF-2α gene expression by quantitative reverse transcription polymerase chain reaction (qRT-PCR) as well as ALDH1A1, LGR5 and β-catenin levels in tissue homogenate by enzyme-linked Immunosorbent assay (ELISA).

### Blood sampling

5 ml of venous blood was withdrawn from all BC patients (before surgery) and from the control group. Blood samples were allowed to clot for thirty minutes then centrifuged for 10 min at about 3000×*g* for separating serum. Serum was removed and stored at – 80 ℃ until used for assaying ALDH1A1, LGR5 and β-catenin levels by ELISA.

### Gene expression analysis of Znhit1 and HIF-2α

Total RNA was extracted from Human BC tissues and adjacent normal breast tissues using RNeasy Mini Kit (Qiagen, Germany). Using a NanoDrop 2000 Spectrophotometer, the concentration of extracted RNA was measured by measuring the absorbance at 260 nm (A260) and RNA purity was determined by using the ratio of absorbance values at 260 nm and 280 nm (A260/A280), A260/A280 ratio of pure RNA is 1.9–2.1. The expression of both Znhit1 and HIF-2α genes was determined by qRT-PCR using RealMOD™ Green qRT-PCR mix Kit according to manufacturer’s protocols. The Real time PCR results were analyzed using the comparative cycle threshold (ΔΔCt) method for calculating fold change of gene expression as the following:

$$ {\text{Ct}}_{{{\text{GOI}}}} \left( {{\text{Sample}}} \right) \, - {\text{ Ct}}_{{{\text{Norm}}}} \left( {{\text{Sample}}} \right) \, = \, \Delta {\text{Ct }}\left( {{\text{Sample}}} \right), $$

$$ {\text{Ct}}_{{{\text{GOI}}}} \left( {{\text{Calibrator}}} \right) \, - {\text{ Ct}}_{{{\text{Norm}}}} \left( {{\text{Calibrator}}} \right) \, = \, \Delta {\text{Ct }}\left( {{\text{Calibrator}}} \right), $$

$$ \Delta {\text{Ct }}\left( {{\text{Sample}}} \right) \, - \, \Delta {\text{Ct }}\left( {{\text{Calibrator}}} \right) = \, \Delta \Delta {\text{Ct}}, $$

$$ {\text{Fold change }} = { 2}^{{ - \Delta \Delta {\text{Ct}}}} , $$

where, *GOI*: Genes of Interest (Znhit1 and HIF-2α), *Norm* Normalizer (GAPDH).

### Determination of tissue and serum ALDH1A1 levels

ALDH1A1 levels in serum of all studied individuals and tissue homogenate of BC tissues and adjacent normal tissues were determined by ELISA assay kit according to the manufacturer’s operating instructions (Cloud-Clone, USA). The assay is based on sandwich enzyme-linked immunosorbent technique. The microwells were coated with an anti-ALDH1A1 antibody (primary antibody). Primary antibody adsorbed to the microwells binds to human ALDH1A1 found in the sample or standard. Anti-human ALDH1A1 conjugated to horseradish peroxidase (HRP) was applied to human ALDH1A1 captured by the primary antibody. After incubation, unbound HRP-conjugated anti-human ALDH1A1 antibody was eliminated by washing and substrate solution was added. The formation of colored product was proportional to ALDH1A1 quantity in the sample or standard. By adding acid, the process was stopped and the absorbance (Abs) was read at 450 nm. The standard curve was constructed by graphing the Abs at 450 nm obtained for each of the six standard concentrations on the vertical (Y) axis against the appropriate concentration on the horizontal (X) axis, then the concentration of ALDH1A1 in samples was determined.

### Determination of tissue and serum LGR5 levels

LGR5 levels in serum of all studied individuals and tissue homogenate of BC tissues and adjacent normal tissues were determined by ELISA assay kit according to the manufacturer’s operating instructions (Cloud-Clone, USA). The assay is based on sandwich enzyme-linked immunosorbent technique. The microwells were coated with an anti-LGR5 antibody (primary antibody). Primary antibody adsorbed to the microwells binds to human LGR5 found in the sample or standard. Anti-human LGR5 conjugated to HRP was applied to human LGR5 captured by the primary antibody. After incubation, unbound HRP-conjugated anti-human LGR5 antibody was eliminated by washing and substrate solution was added. The formation of colored product was proportional to LGR5 quantity in the sample or standard. By adding acid, the process was stopped and Abs was read at 450 nm. The standard curve was constructed by graphing the Abs at 450 nm obtained for each of the six standard concentrations on the vertical (Y) axis against the appropriate concentration on the horizontal (X) axis, then the concentration of LGR5 in samples was determined.

### Determination of tissue and serum β-catenin levels

β-catenin levels in serum of all studied individuals and tissue homogenate of BC tissues and adjacent normal tissues were determined by ELISA assay kit according to the manufacturer’s operating instructions (Cloud-Clone, USA). The assay is based on sandwich enzyme-linked immunosorbent technique. The microwells were coated with an anti-β-catenin antibody (primary antibody). Primary antibody adsorbed to the microwells binds to human β-catenin found in the sample or standard. Anti-human β-catenin conjugated to HRP was applied to human β-catenin captured by the primary antibody. After incubation, unbound HRP-conjugated anti-human β-catenin antibody was eliminated by washing and substrate solution was added. The formation of colored product was proportional to β-catenin quantity in the sample or standard. By adding acid, the process was stopped and Abs was read at 450 nm. The standard curve was constructed by graphing the Abs at 450 nm obtained for each of the six standard concentrations on the vertical (Y) axis against the appropriate concentration on the horizontal (X) axis, then the concentration of β-catenin in samples was determined.

### Statistical analysis

The IBM SPSS software program version 20.0 was used to analyze the data. The mean and standard error (SE) were used to describe quantitative data. The Kolmogorov–Smirnov test was used to check the normality distribution of the quantitative variables. The parametric tests were used since the variables have a normal distribution. The independent t-test was used to compare two independent groups, while the paired t-test was used to compare two dependent groups. Pearson coefficient was used to assess correlations between two quantitative variables. The significance of our results was assessed at a 5% level. The receiver operating characteristic (ROC) curve was used to assess the diagnostic performance of the investigated parameters.

### Ethics approval and consent to participate

This study was approved by ethical committee of Medical Research Institute (MRI)-Alexandria University. A written informed consents were taken from all contributors in this study, in accordance with the Declaration of Helsinki.

## Data Availability

The datasets used and/or analyzed during the current study are available from the corresponding author on reasonable request.
